# Bayesian machine learning improves single-wavelength anomalous diffraction phasing

**DOI:** 10.1107/S2053273319011446

**Published:** 2019-10-07

**Authors:** Maria-Jose Garcia-Bonete, Gergely Katona

**Affiliations:** a Department of Chemistry and Molecular Biology, University of Gothenburg, Box 462, Gothenburg, 40530, Sweden

**Keywords:** single-wavelength X-ray anomalous diffraction, SAD, Friedel pairs, Bijvoet pairs, continuous rotation data collection, inverse-beam geometry, Bayesian inference, survivin

## Abstract

Single-wavelength X-ray anomalous diffraction (SAD) is a frequently employed technique to solve the phase problem in X-ray crystallography. The precision and accuracy of recovered anomalous differences are crucial for determining the correct phases. Continuous rotation (CR) and inverse-beam geometry (IBG) anomalous data collection methods have been performed on tetragonal lysozyme and monoclinic survivin crystals and analysis carried out of how correlated the pairs of Friedel’s reflections are after scaling. A multivariate Bayesian model for estimating anomalous differences was tested, which takes into account the correlation between pairs of intensity observations and incorporates the *a priori* knowledge about the positivity of intensity. The CR and IBG data collection methods resulted in positive correlation between *I*(+) and *I*(−) observations, indicating that the anomalous difference dominates between these observations, rather than different levels of radiation damage. An alternative pairing method based on near simultaneously observed Bijvoet’s pairs displayed lower correlation and it was unsuccessful for recovering useful anomalous differences when using the multivariate Bayesian model. In contrast, multivariate Bayesian treatment of Friedel’s pairs improved the initial phasing of the two tested crystal systems and the two data collection methods.

## Abstract 2

The *a posteriori* probability densities of anomalous structure-factor amplitude differences were estimated by the Markov chain Monte Carlo machine-learning method. The model incorporated the correlation between the different Bijvoet pairs and the improved estimates were shown to be beneficial for SAD phasing.

## 1. Introduction   

X-ray crystallography is one of the most frequently used techniques in structural biology to solve molecular structures at the atomic level. It is suitable for a wide range of molecular sizes, starting from a few atoms to many thousands, and has allowed the structures of more than 135 000 macromolecules, such as proteins, to be solved (Berman *et al.*, 2000 ▸).

A typical single-crystal diffraction experiment consists of exposing a rotating crystal to an incident X-ray beam and collecting the relative reflection beam intensities from the recorded diffraction patterns. Reasonably accurate starting phase information is essential for the many steps leading to the final structural model. Molecular replacement is the most commonly used method to determine the structure of biomolecules by X-ray diffraction (Hendrickson, 2014 ▸). However, *de novo* structure determination requires experimental phases.

One of the experimental methods to obtain initial phases is single-wavelength anomalous X-ray diffraction (SAD) (Hendrickson, 1991 ▸). This technique is gaining popularity (Hendrickson, 2014 ▸) and requires atoms with anomalous X-ray diffraction (often heavy atoms), which causes a small difference in the intensities between reflections related by Friedel’s symmetry.

The experimental observations are different diffraction intensities recovered near simultaneously or at different time points. Because of absorption by atoms in the structure, Friedel’s pairs will have different intensity, but this is not the only factor that affects the difference. Radiation damage makes it difficult to determine the anomalous difference and mean non-anomalous intensity. For the purposes of SAD, only the anomalous difference is useful to localize the anomalous scattering atom in the unit cell. For this reason, it is important to reduce radiation damage using cryogenic cooling, optimizing exposure time and beam intensity.

Friedel’s pairs are not observed simultaneously. It is frequently assumed that decreasing the time between recording one of the Friedel’s pair and the other improves the anomalous data quality. This led to the development of the inverse-beam geometry (IBG) method (Hendrickson *et al.*, 1985 ▸; Dauter, 1997 ▸; Rice *et al.*, 2000 ▸). According to de Sanctis *et al.* (2016 ▸), a systematic study on the beneficial use of IBG is not available, but there are numerous reports where IBG was used successfully (Liu *et al.*, 2012 ▸, 2013 ▸; Akey *et al.*, 2014 ▸; Jungnickel *et al.*, 2018 ▸; Noble *et al.*, 2018 ▸; Rozov *et al.*, 2019 ▸). An alternative strategy focuses on increasing the occurrence of Bijvoet’s pairs on the same diffraction image and thereby minimizing the effect of radiation damage difference and the effect of other time-dependent systematic errors between them (Dauter, 1999 ▸).

Diffraction intensities are recorded as a difference between integrated Bragg peaks and the surrounding diffuse background. As the Bragg peaks become weaker at higher diffraction angles, the level of diffuse scattering can occasionally be higher than that of the Bragg peak, leading to negative recorded intensity. In serial femtosecond crystallography data, even at low-angle, strong reflections have a substantial fraction of negative intensity observations (Sharma *et al.*, 2017 ▸). Negative intensities have no place in diffraction theory; therefore, French and Wilson developed an ingenious Bayesian treatment for these reflections (French & Wilson, 1978 ▸). This method represents the first use of Bayesian statistics in physical sciences and it is still widely used in programs such as *truncate* (French & Wilson, 1978 ▸), *ctruncate* (Zwart, 2005 ▸; Dauter, 2006 ▸) and *XDSCONV* (Kabsch, 2010 ▸). French & Wilson’s method was adapted to the interpretation of just a single set of observations (univariate model). More recently, we suggested a multivariate method based on Bayesian statistical modelling and probabilistic machine learning (Salvatier *et al.*, 2016 ▸). Using Markov chain Monte Carlo (Gilks *et al.*, 1995 ▸) sampling, we model the joint probability of two reflection intensities in order to yield more accurate differences between the underlying structure-factor amplitudes and to determine the uncertainty of differences (multivariate model) (Katona *et al.*, 2016 ▸). This method retains the most important *a priori* belief about diffraction intensities: they cannot be negative (Katona *et al.*, 2016 ▸). The main difference between the univariate and multivariate treatment of difference structure-factor amplitudes is that the latter incorporates the concept of covariance (correlations) between the paired observations. Correlation is often overlooked and arises from the fact that the two measurements are not independent. For example, two non-independent diffraction measurements can be made on the same crystal volume in the forward and the reverse direction. This volume will share the same molecules, with their characteristic mosaic misalignments, crystal defects *etc*. If the experiments and comparisons are done carefully, correlations will arise naturally and it is counterproductive to make efforts to eliminate them.

It is important to emphasize that the multivariate treatment does not substitute scaling procedures in its current form. Standard crystallographic scaling protocols for example in *HKL2000* (Otwinowski & Minor, 1997 ▸), *XDS* (Kabsch, 2010 ▸), *SCALA/AIMLESS* (Evans, 2011 ▸; Evans & Murshudov, 2013 ▸) software take into account many of the systematic errors affecting data collection. It is also recognized that matching of equivalent reflections could be beneficial for anomalous scaling purposes (local scaling), which is implemented for example in the *Madsys* suite (Hendrickson, 1991 ▸). Many scaling methods, including local scaling, can be used in conjunction with the multivariate Bayesian method. The point estimate of the scaled, unmerged intensity is treated as a fixed observation and these observations are the starting point for pairing and multivariate analysis. The errors of the scaling model are taken into account by the covariance matrix together with the experimental variations.

Theoretically, the univariate, standard treatment of diffraction intensities is only correct if the measurements are truly independent and there is no correlation between the measurements. Fortunately, the *maximum likelihood* estimate of the mean parameter is the same with and without taking correlations into account. From this it also follows that the difference between the means is identical at any level of correlation. But, the uncertainty of the estimates will strongly depend on the correlation. The higher the correlation, the narrower the confidence interval of the difference estimate will get; in other words, high correlation in the observations yields more precise difference estimates. The uncertainty of anomalous difference measurements is important information for phasing algorithms when they rank the potential solutions. Unfortunately, not only the uncertainty estimates are affected: the univariate French/Wilson and our multivariate method use an *a posteriori* rather than a *maximum likelihood* estimate, which makes the univariate and multivariate methods not interchangeable at all. This is especially apparent when comparing weak reflections.

Although the multivariate method was shown to work well on synthetic data (Katona *et al.*, 2016 ▸), experimental data often contain unexpected contributions of systematic and random errors. Here, we test the multivariate Bayesian method on a well known experimental problem of crystallography where success or failure can be confidently evaluated.

**Figure 1:**
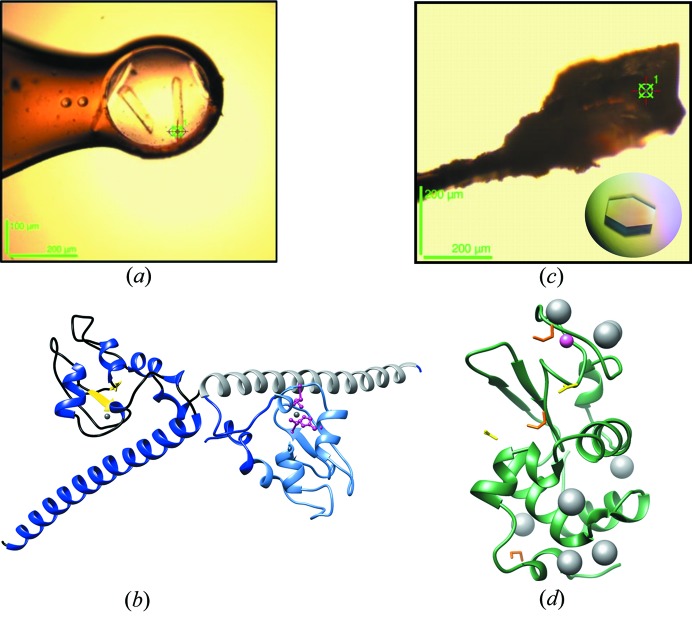
(*a*) Rod-like survivin crystal with approximate dimensions 150 × 30 × 30 µm. (*b*) Survivin dimer structure obtained from the CR multivariate analysis. In the left monomer, the dark blue colour represents the α-helices including the long C-terminal α-helix and yellow colour the β-sheets. In the right monomer, the BIR domain is shown in light blue and the C-terminal domain in grey. The Zn^2+^ ions are represented as a grey sphere in the BIR domains. In the right monomer, the coordinating amino acids are represented in pink (Cys-57, Cys-60, His-77 and Cys-84). (*c*) Tetragonal lysozyme crystal with approximate dimensions of 200 × 200 × 200 µm. (*d*) Lysozyme structure obtained from the CR multivariate analysis containing a sodium ion represented as a pink sphere, eight chloride ions represented as grey spheres, three ethyl­ene glycol molecules in orange and two acetate ions in yellow.

The test proteins used in this study are human survivin and hen egg-white lysozyme. In order to test the influence of moderate resolution, small, elongated survivin crystals were used [Fig. 1 ▸(*a*)]. Survivin is a small human protein of 16.5 kDa and is a member of the Inhibitor Apoptosis Protein (IAP) family involved in cell cycle division and in apoptosis (Sun *et al.*, 2005 ▸). In the Baculovirus Inhibitor of Apoptosis (BIR) domain, a Zn^2+^ ion is coordinated by cysteine and histidine residues [Fig. 1 ▸(*b*)] and the wavelength was optimized for the element zinc in the anomalous X-ray diffraction experiment. While zinc has relatively strong anomalous signal at 9.66 keV, these crystals diffracted weakly, reaching only to 3.2 Å resolution.

Lysozyme forms well diffracting crystals [Fig. 1 ▸(*c*)], which contain sulfur atoms natively in eight cysteine and two methio­nine amino acid residues. In addition, the solvent region contains eight chloride ions at well defined positions in the unit cell [Fig. 1 ▸(*d*)] (Evans & Bricogne, 2002 ▸). These two elements yielded weak anomalous diffraction when irradiated with 8 keV X-rays. These crystals also have a sodium ion which was described previously in other lysozyme structures, such as Protein Data Bank (PDB) entries 5apd (Lundholm *et al.*, 2015 ▸) and 193l (Vaney *et al.*, 1996 ▸). The sodium ion is stabilized by the Ser60–Leu75 loop.

These two crystal systems represent different SAD phasing scenarios. Survivin crystals are weakly diffracting and have lower symmetry, but they have potentially stronger anomalous signal due to the presence of Zn^2+^. They also have 69% solvent content, which tends to facilitate density modifications and SAD phasing. The properties of lysozyme crystals are diagonally opposite: strong diffraction and high symmetry, but they contain weak anomalous scatterers and their solvent content is lower at 41%.

In this work, we analysed continuous rotation (CR) and IBG collection methods for two different protein crystals (lysozyme and survivin) with different symmetry and resolution. We were interested in determining the best way of pairing anomalous reflections and whether or not structure-factor calculations based on a multivariate Bayesian model improve experimental phasing in practice.

## 2. Methods   

### 2.1. Protein purification and crystallization   

Tetragonal lysozyme crystals [Fig. 1 ▸(*c*)] were grown according to the method described previously (Lundholm *et al.*, 2015 ▸), using lyophilized hen egg-white lysozyme (Sigma–Aldrich, St Louis, Missouri, USA). The crystals were cryo-cooled in liquid nitro­gen.

Survivin was expressed in *Escherichia coli* and purified according to the protocol described previously (Garcia-Bonete *et al.*, 2017 ▸). The His-tag was removed by thrombin digestion and a subsequent gel-filtration purification step. The crystallization conditions were discovered in the PACT crystallization screening (Molecular Dimensions) using a Mosquito LCP robot (TTPLabtech). The protein was concentrated in a buffer containing 50 m*M* Tris pH 8.0, 150 m*M* NaCl and 1 m*M* DTT (dithiothreitol) to 20 mg ml^−1^ and mixed 1:1 with the precipitation solution (0.2 *M* sodium citrate pH 6.5, 0.1 *M* bistris propane and 20% PEG 3500). The crystals were grown using the sitting-drop vapour diffusion method at room temperature (20°C). The rod-like crystals [Fig. 1 ▸(*a*)] appeared in approximately 1–3 days and were cryo-cooled in liquid nitro­gen using 20% glycerol as additional cryoprotectant on micro-loops (MiTeGen).

### 2.2. X-ray diffraction data collection   

The data were collected at beamline ID30B at the European Synchrotron Radiation Facility (ESRF, Grenoble, France). This beamline is a tunable-wavelength end-station, which covers the energy range of 6–20 keV (de Sanctis *et al.*, 2016 ▸; McCarthy *et al.*, 2018 ▸). The zinc X-ray absorption edge was identified by an X-ray fluorescence scan performed prior to the diffraction data collection. The beamline is equipped with a DECTRIS Pilatus 6M-F detector and an MD2-S X-ray Microdiffractometer. The diffractometer has a maximum speed of 720° s^−1^ (dynamic precision 2.3 mdeg). The maximum speed was 120° s^−1^ during the experiment when the crystal was reoriented during IBG data collection. At this speed, the dynamic precision is approximately 0.3 mdeg. The sphere of confusion at kappa 0° is 1 µm at the maximum speed. The X-ray beam is controlled by a vacuum-compatible rotary shutter developed at the ESRF. The shutter is controlled by the MD2-S control software and it is synchronized to the detector with an accuracy of 1 to 3 ms.

**Figure 2:**
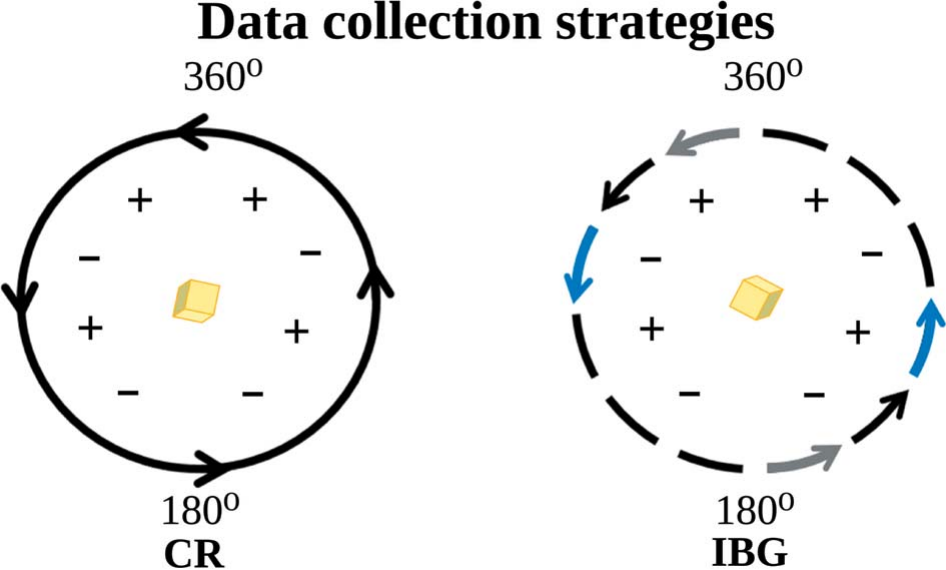
Comparison of CR and IBG collection approaches. The crystal is represented in yellow and the *I*(+) and *I*(−) reflections as plus and minus symbols.

Two different types of data aquisition were tested: CR and IBG data collection (Fig. 2 ▸). The CR data collection consisted of collecting 360° rotation range in a single sweep. During IBG data collection, after every 10° rotation wedge the crystal was reoriented by a 180° rotation and a complementary inverse rotation wedge was collected containing the Friedel’s pairs of the first wedge. In both cases, the crystals were initially randomly oriented. The IBG collection was performed using the beamline control software to assign starting angles, image and run numbering for each data set. The data collection parameters were determined with the help of *EDNA* software (Incardona *et al.*, 2009 ▸). The individual data sets and the estimated absorbed doses are summarized in Table S1 in the supporting information. The absorbed dose was estimated with the program *RADDOSE-3D* (Bury *et al.*, 2018 ▸). *R*_D_ values for the individual data sets were calculated with the program *XDSSTAT* (Diederichs, 2006 ▸) and they are plotted in Figs. S1, S2 and S3.

The X-ray wavelengths were adjusted according to the different scattering elements in the survivin and lysozyme crystals, Zn [λ = 1.273 Å (9.66 keV)] and S, Na and Cl [λ = 1.550 Å (8.00 keV)], respectively. The beam size was always the same, 50 µm (horizontal) × 30 µm (vertical), and the estimated flux after attenuation is listed in Table S1. The X-ray beam in all cases intersected the spindle axis.

X-ray diffraction data were collected from four survivin crystals (two CR and two IBG collections) and nine lysozyme crystals (four CR and five IBG collections).

### 2.3. Univariate treatment of reflection intensities   

The IBG data wedges were pooled into two complementary data sets and were processed separately using the *XDS* package (Kabsch, 2010 ▸). The data from the half rotations of all crystals were scaled together using *XSCALE* (Kabsch, 2010 ▸). For univariate data reduction, X-ray diffraction images from CR and IBG merging were also treated by the program *XDSCONV* (Kabsch, 2010 ▸). The resolution was restricted to 3.2 Å and 1.61 Å for the survivin and lysozyme diffraction data, respectively.

### 2.4. Pairing of anomalous reflections and multivariate Bayesian machine learning   

Unmerged reflections were generated by *XDS* and were scaled using *XSCALE*. The pairing of reflections was based on two criteria: either by their direct matching of Friedel’s pairs (up to 1° difference in IBG mode or 180° rotation ± 1° difference in CR mode) or by attempting to pair Bijvoet’s pairs up to 10° in the CR mode. If there were no valid pairs found, the reflection intensity was treated as a univariate observation. With every observation, pairing was attempted with any complementary observations. This was performed in two sweeps: first collecting *I*(−) complementary pairs of *I*(+) observations, then the complementary *I*(+) pairs of *I*(−) reflections. Once the best of the acceptable *I*(+)–*I*(−) pairing was found, both observations were flagged and they were never reused. After the two pairing sweeps, the still unpaired reflections were collected in unpaired *I*(+) and *I*(−) categories in addition to the naturally unpaired centric reflections.

The pairing of acentric anomalous reflections was performed by an algorithm developed in the Python programming language with the libraries *cctbx*, *pandas*, *NumPy* and *SciPy*. *cctbx* and *iotbx* packages were used to map the scaled, unmerged, anomalous reflections to the asymmetric unit of the corresponding space groups of survivin (*C*2) and lysozyme (*P*4_3_2_1_2) crystals. The *pandas* library was used to store and search among the large number of paired and unpaired reflection data obtained from the different crystals and data collection methods.

### 2.5. Bayesian machine learning of structure-factor amplitudes and their anomalous difference   

The paired intensities were the basis of the Bayesian multivariate machine learning protocol developed with the help of the *pymc3* library (Salvatier *et al.*, 2016 ▸). The *a priori* distribution of the structure-factor amplitudes was assumed to be uniform (from 0 to 10^8^). If the number of reflection pairs was more than or equal to five, then the paired anomalous reflections were analysed according to the method described previously (Katona *et al.*, 2016 ▸) and any additional unpaired reflections were ignored. LKJ log-likelihood (ν = 1) (Lewandowski *et al.*, 2009 ▸) and the lognormal (μ = 0, σ = 1) *a priori* distributions were used to generate the correlation matrix and individual variances, respectively. The covariance matrix of the multivariate normal distribution was calculated as described previously (Barnard *et al.*, 2000 ▸). While the addition of unpaired observations (assuming univariate distribution) could improve the mean and variance estimates of *F*(+) or *F*(−), frequent numerical instabilities were observed during Markov chain Monte Carlo (MCMC) (Gilks *et al.*, 1995 ▸) sampling when using mixed univariate/multivariate likelihoods. This limitation forced us to abandon the univariate fraction of the reflections. Very few reflections were lost in the Friedel’s pairing mode in this way. Successful pairing is less probable when targeting Bijvoet pairs observed in rapid succession, especially when starting with randomly oriented crystals. This resulted in a substantial drop in effective multiplicity when multivariate Bayesian inference was used in the Bijvoet pairing mode. If the number of reflection pairs was less than five, the pairs were split up and added to the unpaired *I*(+) and *I*(−) reflections. They were treated as independent, univariate, truncated normal distributions. Centric reflections, *I*(+) with no corresponding *I*(−) observations and *I*(−) with no corresponding *I*(+) observations, were always estimated as univariate, truncated normal distributions and no anomalous differences were estimated. Empirical Bayes priors such as Wilson’s were not used when treating the reflections with a non-standard method (Wilson, 1949 ▸). For all models, the MCMC sampling (Gilks *et al.*, 1995 ▸) was performed with the Metropolis stepping method (Metropolis *et al.*, 1953 ▸). Of the 100 000 total samples, the first 90 000 were discarded. The density of the posterior probability distribution was estimated using a multivariate kernel density estimation method for visualization purposes (Parzen, 1962 ▸).

The multivariate analysis was not performed on the Bijvoet pairing of the survivin data set due to the low multiplicity in the CR or IBG data. However, because of the high symmetry of lysozyme crystals and high multiplicity of reflections, it was also possible to analyse the Bijvoet pairing using multivariate analysis.

From the multivariate analysis, two files were generated, one log file and one structure-factor file (*hkl*), mimicking the *CCP4* (F,SigF,DF,SigDF,isym) output format of *XDSCONV*. The log file contained diagnostic information regarding the multivariate Bayesian analysis and the *hkl* file contained the structure-factor and anomalous difference information necessary for further analysis. Bayesian analysis of approximately 1% of the reflections did not converge, generating an improbably high correlation parameter, high autocorrelation and drifting in the MCMC parameter traces. These reflections were reprocessed and, if the analysis repeatedly failed to converge, they were discarded from further analysis.

### 2.6. Phasing, model building and refinement of the structures   

The data file was transformed to a structure-factor file (mtz) using the *F2MTZ* and *Cad* utility and an *R*_free_ flag was generated using the program *Sftools* of the *CCP4* package (Winn *et al.*, 2011 ▸). The phases were obtained using default options of *Autosol* (Terwilliger *et al.*, 2009 ▸) in the *PHENIX* suite by selecting the most relevant scattering element for each protein (Zn for survivin and S for lysozyme) and the number of molecules per asymmetric unit was estimated according to the Matthews coefficient calculated by *Xtriage* (Table 1 ▸). The anomalous peak heights are summarized and compared in Table S2. For initial model building, *Autobuild* (Terwilliger *et al.*, 2008 ▸) was used and the resulting initial model was manually rebuilt and refined using *Coot* (Emsley & Cowtan, 2004 ▸) and *phenix.refine* (Afonine *et al.*, 2012 ▸), respectively.

### 2.7. Correlation between the paired reflections   

The paired intensities were also the basis of the maximum likelihood Pearson coefficient calculations using the library *SciPy*. Only reflections with more than five observation pairs were included in this analysis and the correlation between *I*(+) and *I*(−) values was determined. The *d* spacing (Å) was calculated using *cctbx* and *uctbx* packages. The resolution binning used to plot the Pearson correlation results was manually selected to keep similar bin sizes and to show how the correlation differs at higher resolution for each protein. Pearson correlation coefficients were calculated for the two data collection methods in each protein, except for the Bijvoet pairing of CR survivin diffraction data, which was not possible due to the low reflection multiplicity.

## 3. Results and discussion   

### 3.1. Comparing the correlation of paired observations of IBG versus CR collection   

**Figure 3:**
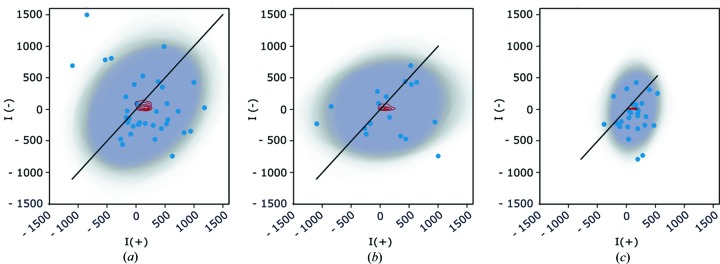
Distribution of intensity observations of a single reflection for lysozyme crystal structure (36, 18, 8). (*a*) Friedel pairing of CR data. (*b*) Bijvoet pairing of CR data. (*c*) Friedel pairing of IBG data. *I*(+) and *I*(−) observation pairs are represented as blue dots and posterior density of the mean [*F*(+)^2^, *F*(−)^2^] location parameters of the multivariate normal distribution is represented by red contour lines (arbitrary units). Each MCMC sample of the joint posterior distribution is represented by a transparent 95% isodensity ellipse, which gradually adds up to darker and darker grey. The length and direction of the ellipse axes are determined from the diagonalized covariance matrix of each MCMC trace sample.

**Figure 4:**
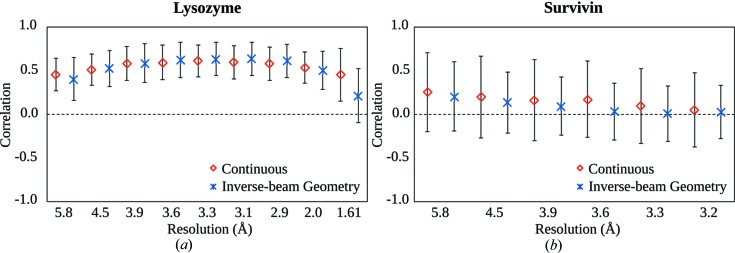
Pearson correlation coefficient between the Friedel’s pairs of reflections of lysosyme (*a*) and survivin (*b*). The mean and standard deviation of all unique acentric reflections that belong to the same resolution bins are displayed. Red and blue represent the CR data and the IBG data, respectively.

Fig. 3 ▸ represents a weak reflection (*h* = 36, *k* = 18, *l* = 8) of lysozyme for Friedel pairing [Fig. 3 ▸(*a*)] and Bijvoet pairing [Fig. 3 ▸(*b*)] of CR data. The Friedel pairing of IBG data is shown in Fig. 3 ▸(*c*). This example illustrates that Friedel pairing typically resulted in a greater number of observation pairs than Bijvoet pairing and therefore the posterior estimate of means is better defined. When comparing Friedel pairing of CR [Fig. 3 ▸(*a*)] and IBG data [Fig. 3 ▸(*c*)], both have a high number of *I*(+)–*I*(−) pairs; however IBG appears to be less correlated than the CR data. This is in line with the general tendency shown in Fig. 4 ▸ where CR data have a higher correlation than the IBG data. The elongated elipses in Figs. 3 ▸(*b*) and 3 ▸(*c*) are the consequence of different scale parameters in the multivariate distributions of *F*(+)^2^ and *F*(−)^2^ rather than correlation. The intensity difference due to anomalous diffraction is represented by the distance of the posterior density of the means from the diagonal. In practice, the mean and standard deviation of posterior samples of *F*(+)–*F*(−) is reported as the DANO and SIGDANO columns, respectively. All panels in Fig. 3 ▸ show that *F*(+)^2^ is slightly higher than *F*(−)^2^; in Fig. 3 ▸(*b*) the posterior distribution of the means is less defined, whereas in Fig. 3 ▸(*c*) the uncertainty of the anomalous difference is the best of three. In this weak reflection, the number of negative observations is high, and the posterior density of means is also more complex. The density is concentrated above the *x* axis due to the zero *a priori* probability of negative *F*(−) amplitudes, but the peak (and mean) of the density is well separated from the diagonal. This results in a large anomalous difference even for this weak reflection.

Fig. 4 ▸ shows the maximum likelihood point estimates of the Pearson correlation coefficients (CC) between *I*(+)–*I*(−) pairs. When the pairing principle was based on direct Friedel’s pairs [Fig. 4 ▸(*a*)] generally higher correlation between *I*(+) and *I*(−) pairs was observed and the choice of data collection mode had little influence. Friedel’s pairs in survivin data [Fig. 4 ▸(*b*)] show less correlation than in lysozyme data. It is surprising how small an influence radiation damage (at this attenuation level) has on the CC. Between Friedel’s pair *I*(+) and *I*(−) [or *I*(−) and *I*(+)] observations, up to 18 times more X-ray dose is deposited in the CR mode than in the IBG mode. Assuming that X-ray radiation typically decreases diffraction intensity, this effect alone would lead to a decrease in the correlation as it could be either a positive or a negative influence on the anomalous difference, depending on which of the Friedel’s pair is observed first: *I*(+) or *I*(−). It is important to note that we used scaled reflections and scale factors tend to compensate for the general decrease in intensity due to radiation damage; nevertheless a similar argument is valid for site-specific radiation damage.

Survivin Friedel’s pairs are slightly less correlated in the IBG data than in the CR data, but this small difference can be attributed to the uneven sampling of the crystals with differing quality. Towards higher resolution, CC decreases both for the lysozyme and survivin data sets, most likely because counting errors become more important and they tend to be uncorrelated.

In Table S3 and Fig. S4, the different treatments for CR data are compared. Bijvoet’s pairing of lysozyme CR data shows a substantially lower CC (Fig. S4), indicating that the systematic errors are shared to a lesser degree between (non-Friedel) Bijvoet’s pairs than Friedel’s pairs. This may indicate that systematic errors originating from crystal shape (for example, absorption) are more important for (non-Friedel) Bijvoet’s pairs or symmetry equivalents are not perfectly equivalent because of pseudosymmetry in the unit cell. Although the comparison of CCs does not directly reveal the anomalous differences between *I*(+) and *I*(−) pairs, it gives an indication of whether or not multivariate treatment and the introduction of a covariance matrix have a chance to improve the inference.

Schiltz *et al.* suggest keeping data unmerged for phasing purposes (Schiltz & Bricogne, 2010 ▸). This strategy aims to facilitate the detection of site-specific radiation damage during the X-ray measurements and using the broken symmetry from the polarization anisotropy of anomalous scattering. The low CC of non-Friedel Bijvoet’s pairs in contrast to the genuine Friedel’s pairs also supports this recommendation (Fig. S4). We also speculate that assuming crystallographic symmetry often disguises subtle pseudosymmetries in crystal systems and reflections may not be truly symmetry equivalent. It may be tempting to keep all symmetry-related reflections separate, but this would result in a multiplicity of one for a full rotation of a single crystal, thus increasing the uncertainty of anomalous difference estimates enormously. Merging data from multiple crystals becomes even more important and multivariate Bayesian analysis could make especially good use of the limited number of observations.

### 3.2. Experimental phasing   

Table 1 ▸ describes the data quality and phasing information about the survivin and lysozyme crystal structure. Survivin data have a lower signal-to-noise ratio and weaker anomalous signal than lysozyme using conventional measures (〈*I*/σ*I*〉, SigAno). This could be rationalized by the fact that the number of merged crystals is higher in lysozyme than in survivin. Lysozyme crystals have higher symmetry than survivin crystals; therefore the multiplicity is higher. An important consequence of the lower signal-to-noise ratio is that the diffraction resolution of survivin data is only 3.2 Å in contrast to 1.6 Å of lysozyme data.

*Autosol* phasing statistics (Table 1 ▸) suggest that CR multivariate analysis works better for both types of crystals since the initial *R* factors are lower than in univariate treatment of CR data or IBG data irrespective of the treatment. The number of fragments is also the lowest in survivin phasing, indicating a better continuity of the main-chain electron density. For lysozyme phasing all methods found a single fragment. The largest number of amino acids was also found by the multivariate treatment of CR data in the lysozyme system, thus providing slightly better statistics than the univariate treatment of the same CR data. In the survivin system, most amino acids were detected in the univariate treatment of IBG data, but this solution has much worse *R* factors, indicating that many of the numerous (34) peptide fragments were probably built into noise electron density. The number of sites is also closest to the expected number in the multivariate treatment of CR data in both protein systems. The initial phase errors are consistently lower when the data are treated by the multivariate protocol (both for CR and IBG). Among the lysozyme structures the final phase error is also lower when refined against multivariate Bayesian data (both for CR and IBG). The figure of merit was the highest in the multivariate treatment of survivin CR data, but among the lysozyme data sets the highest figure of merit was associated with the univariate treatment of IBG data. Since the number of amino acids was lower in this case, the better figure of merit may simply be the result of a more incomplete, but better defined model.

When we also consider the Bijvoet pairing of the lysozyme CR data, the phasing failed completely with *R* factors greater than 50% and resulted in an uninterpretable experimental electron density (Table S3 and Fig. S4). Such dramatically worse phasing could result from the reduced multiplicity of Bijvoet phasing, but more importantly by focusing on the non-Friedel Bijvoet’s pairs we also enhance any differences due to pseudosymmetry, possibly at the expense of anomalous differences.

**Figure 5:**
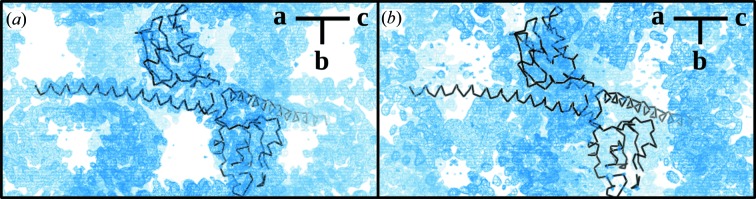
Comparison of electron-density maps after density modification in *Autosol* (Terwilliger *et al.*, 2009 ▸) of the CR survivin data using multivariate (σ = 1.0, ρ = 0.26 e Å^−3^) (*a*) and univariate (σ = 1.0, ρ = 0.29 e Å^−3^) (*b*) analysis. Multivariate analysis of CR data gives the most accurate electron-density map. The figure was generated by the program *PyMOL* (Schrodinger, 2015 ▸) by extending the initial electron-density map of CR multivariate (*a*) and univariate (*b*) analysis with fast Fourier transform software (*fft*) from the *CCP4* package (Winn *et al.*, 2011 ▸). Both images are equally oriented and visualized in an orthoscopic view. The survivin structural model (black) corresponds to the final refined model obtained from the CR multivariate data set.

**Figure 6:**
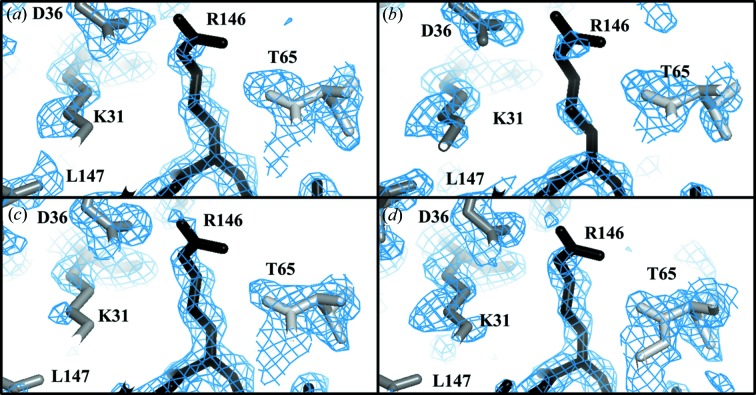
Comparison of electron-density maps after density modification in *Autosol* (Terwilliger *et al.*, 2009 ▸) of the R147 residue from lysozyme data sets. (*a*) and (*b*) correspond to the CR data set treated by the multivariate (σ = 1.0, ρ = 0.48 e Å^−3^) and univariate (σ = 1.0, ρ = 0.48 e Å^−3^) methods, respectively. (*c*) and (*d*) correspond to the IBG data set using multivariate (σ = 1.0, ρ = 0.45 e Å^−3^) and univariate (σ = 1.0, ρ = 0.45 e Å^−3^) analysis, respectively. The figure was generated by the program *PyMOL* (Schrodinger, 2015 ▸) by setting up the r.m.s.d. (root mean square deviation) level to 1.0 and is visualized in an orthoscopic view. The extra electron density surrounding R147 (black) corresponds to symmetry-related molecules (grey).

In the case of survivin diffraction, the contrast between CR multivariate analysis and the other approaches was the greatest. Fig. 5 ▸ shows the initial electron-density maps after density modification as calculated by *Autosol*. Although the same set of starting observations was used, multivariate analysis provided a more continuous electron-density map of the C-terminal α-helix of survivin and fewer noise peaks in the solvent than a univariate approach. It is more difficult to appreciate the improvements of the electron-density maps in lysozyme data sets, but specific amino acids, such as R147 at the C-terminal, show better initial electron density derived from the multivariate analysis [Figs. 6 ▸(*a*), 6 ▸(*c*)] in comparison with univariate analysis [Figs. 6 ▸(*b*), 6 ▸(*d*)].

### 3.3. Refinement   

The different structures were refined using *phenix.refine* and the statistics of refinement are shown in Table 2 ▸. The initial automatically built structures derived from the lysozyme data sets were straightforward to manually rebuild and refine using the experimental maps. In contrast, experimental phases of the survivin data sets were less accurately determined. After the unsuper­vised *Autobuild* procedure of *PHENIX*, the structure of the survivin dimer was only recognizable when the multivariate CR data set was used. Only this data set was used for further manual rebuilding and refinement. When the heavy-atom locations from the multivariate CR protocol were transferred to other data sets, phasing was also successful, indicating that the multivariate protocol assists the crucial heavy-atom search step the most.

Regarding the number of sites found during experimental phasing, in all cases there were more sites detected than expected. In the lysozyme crystal structure, the expected number of sulfur atoms was ten, corresponding to eight cysteine and two methio­nine amino acid residues in the sequence range 19–147. In contrast, *Autosol* detected 18 and 20 sites in the CR and IBG data sets, respectively. The type of data treatment did not affect the number of sites in the case of lysozyme. The remaining heavy atoms were found in the solvent. These were unlikely to be sulfur, because the precipitant solution did not contain any sulfur-containing compounds. In spite of that, chloride has similar *f*′ and *f*′′ at 8 keV X-ray photon energies and the crystallization condition contained sodium chloride. Eight of the extra heavy-atom sites were changed to chloride in all the structures, the rest were removed from the model due to poor electron density. The most accurate number of sites was found when the anomalous differences and structure-factor amplitudes were generated from the CR data set. Published lysozyme structures also show the presence of chloride ions in the solvent, such as PDB entries 1gwd (seven chlorides) (Evans & Bricogne, 2002 ▸) and 1dpx (two chlorides) (Weiss *et al.*, 2000 ▸).

In the survivin crystal structure, only two zinc ions were expected, one for each BIR domain. However, *Autosol* found three to ten sites and this could explain why *Autosol* could not always determine accurate phases. The multivariate treatment of CR survivin data yielded the most accurate estimate of the number of sites (three) when using *Autosol*. In this case, two of the Zn^2+^ ions were correctly placed, but even in this case the third one was not supported by the electron density and was removed by the automated model building and refinement steps.

## 4. Conclusions   

The main conclusion of this work is that Friedel’s pair observations are not independent and it is important to take into account the correlations between measurements as this can improve phasing. Multivariate analysis of the same data consistently outperformed the univariate analysis. We observed the largest contrast between the two methods when their performance was compared on weak diffraction data. Multivariate Bayesian machine learning uses more computational resources, but this is still favourable compared to improving crystal quality and collecting new data when working with a difficult protein system. With weak data, exemplified by our survivin data sets, multivariate treatment of Friedel’s pairs may make the difference between a solved and unsolved structure.

## Supplementary Material

Additional figures and tables. DOI: 10.1107/S2053273319011446/ae5069sup1.pdf

PDB reference: human survivin, 6sho

PDB reference: hen egg-white lysozyme, continuous rotation data collection and multivariate analysis of Friedel pairs, 6sij

PDB reference: hen egg-white lysozyme, continuous rotation, univariate, 6sik

PDB reference: hen egg-white lysozyme, inverse-beam geometry data collection and multivariate analysis of Friedel pairs, 6sil

PDB reference: hen egg-white lysozyme, inverse-beam geometry, univariate, 6sim

## Figures and Tables

**Table:** 

	Survivin		Lysozyme	
	CR	IBG	CR	IBG
No. merged data sets	2	2	5	4
Space group	*C*2	*C*2	*P*4_3_2_1_2	*P*4_3_2_1_2
Cell dimensions				
*a*, *b*, *c* (Å)	115.0, 71.2, 81.3	115.0, 71.2, 81.3	78.9, 78.9, 36.9	78.9, 78.9, 36.9
α, β, γ (°)	90.0, 127.9, 90.0	90.0, 127.9, 90.0	90.0, 90.0, 90,0	90.0, 90.0, 90,0
Wavelength (Å)	1.27265	1.27265	1.54980	1.54980
Resolution (Å)	14.31–3.20 (3.28–3.20)	14.31–3.20 (3.28–3.20)	7.20–1.61 (1.65–1.61)	7.20–1.61 (1.65–1.61)
Observed reflections	112872 (8392)	225601 (16986)	1438053 (21401)	1075082 (4675)
Unique reflections	16783 (1248)	16738 (1227)	28771 (2102)	28213 (1392)
Multiplicity	6.7 (6.7)	13.5 (13.8)	39.2 (5.8)	38.1 (3.4)
Completeness (%)	99.1 (99.1)	99.1 (98.6)	99.9 (99.2)	97.3 (65.7)
*R*_merge_ (%)	12.2 (79.5)	24.1 (143.6)	7.9 (22.3)	6.4 (27.7)
〈*I*/σ*I*〉	12.04 (2.03)	7.53 (0.92)	47.53 (8.84)	44.83 (3.19)
CC(1/2) (%)	99.6 (78.7)	99.7 (81.3)	100 (98.5)	100 (91.5)
Anomalous correlation (%)	24 (−7)	21 (12)	54 (49)	46 (26)
SigAno	1.03 (0.70)	1.03 (0.71)	1.56 (1.10)	1.43 (1.05)

**Table:** 

	Survivin				Lysozyme			
	CR	CR	IBG	IBG	CR	CR	IBG	IBG
Analysis	Univariate	Multivariate	Univariate	Multivariate	Univariate	Multivariate	Univariate	Multivariate
*R*_work_ (%)	46.80	41.88	47.95	46.15	19.07	18.60	20.82	19.76
*R*_free_ (%)	53.45	45.06	53.45	52.99	20.26	20.14	23.51	21.51
No. fragments	14	12	34	14	1	1	1	1
No. amino acids	112	136	163	118	125	127	122	124
Water	0	0	0	0	147	138	144	140
Sites	4	3	10	5	18	18	20	20
Figure of merit	0.24	0.26	0.25	0.21	0.48	0.47	0.49	0.48
Initial phase error (°)	59.84	54.63	59.89	61.14	20.00	19.71	24.57	21.86
PDB entry		6sho			6sik	6sij	6sim	6sil

**Table 2:** Refinement statistics for survivin and lysozyme structures RMS = root mean square.

	Survivin	Lysozyme			
	CR	CR	CR	IBG	IBG
Analysis	Multivariate	Univariate	Multivariate	Univariate	Multivariate
*R*_work_ (%)	20.08	13.53	14.06	14.60	14.35
*R*_free_ (%)	25.88	16.27	16.73	17.38	16.90
RMS bond lengths (Å)	0.01	0.01	0.01	0.01	0.01
RMS bond angles (°)	1.18	0.81	0.83	0.78	0.79
Average *B* factors (Å^2^)	106.67	16.06	15.03	18.92	18.30
Ramachandran outliers (%)	0	0	0	0	0
Ramachandran favoured (%)	93.23	98.43	98.43	98.43	98.43
Rotamer outliers (%)	0	0	0	0	0
Clashscore	13.79	2.85	1.90	4.27	3.28
Final phase error (°)	33.72	15.96	15.20	18.58	16.12
PDB entry	6sho	6sik	6sij	6sim	6sil
