# Supplementary material for: Bayesian machine learning improves single-wavelength anomalous diffraction phasing
Source: Acta Crystallogr A Found Adv. 2019 Oct 7;75(Pt 6):851–60. doi: 10.1107/S2053273319011446 (PMC6833979; doi:10.1107/S2053273319011446)
Supplement: Supplementary file 1 [file a-75-00851-sup1.pdf]

FOUNDATIONS
ADVANCES

**Volume 75 (2019)**

**Supporting information for article:**

# Bayesian machine learning improves single-wavelength anomalous diffraction phasing

**Maria-Jose Garcia-Bonete and Gergely Katona**

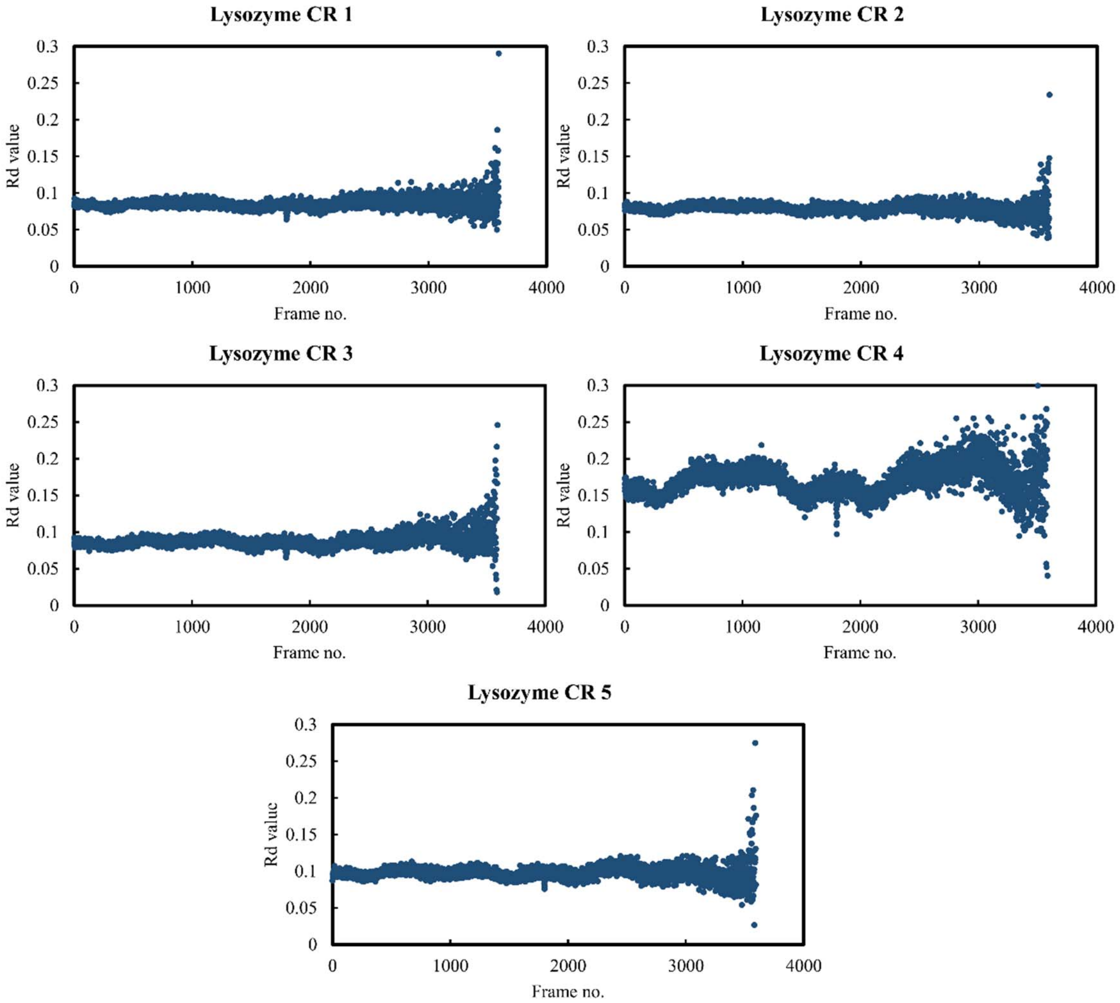


**Figure S1.** $R_D$ plots of the individual data sets of lysozyme CR collection.

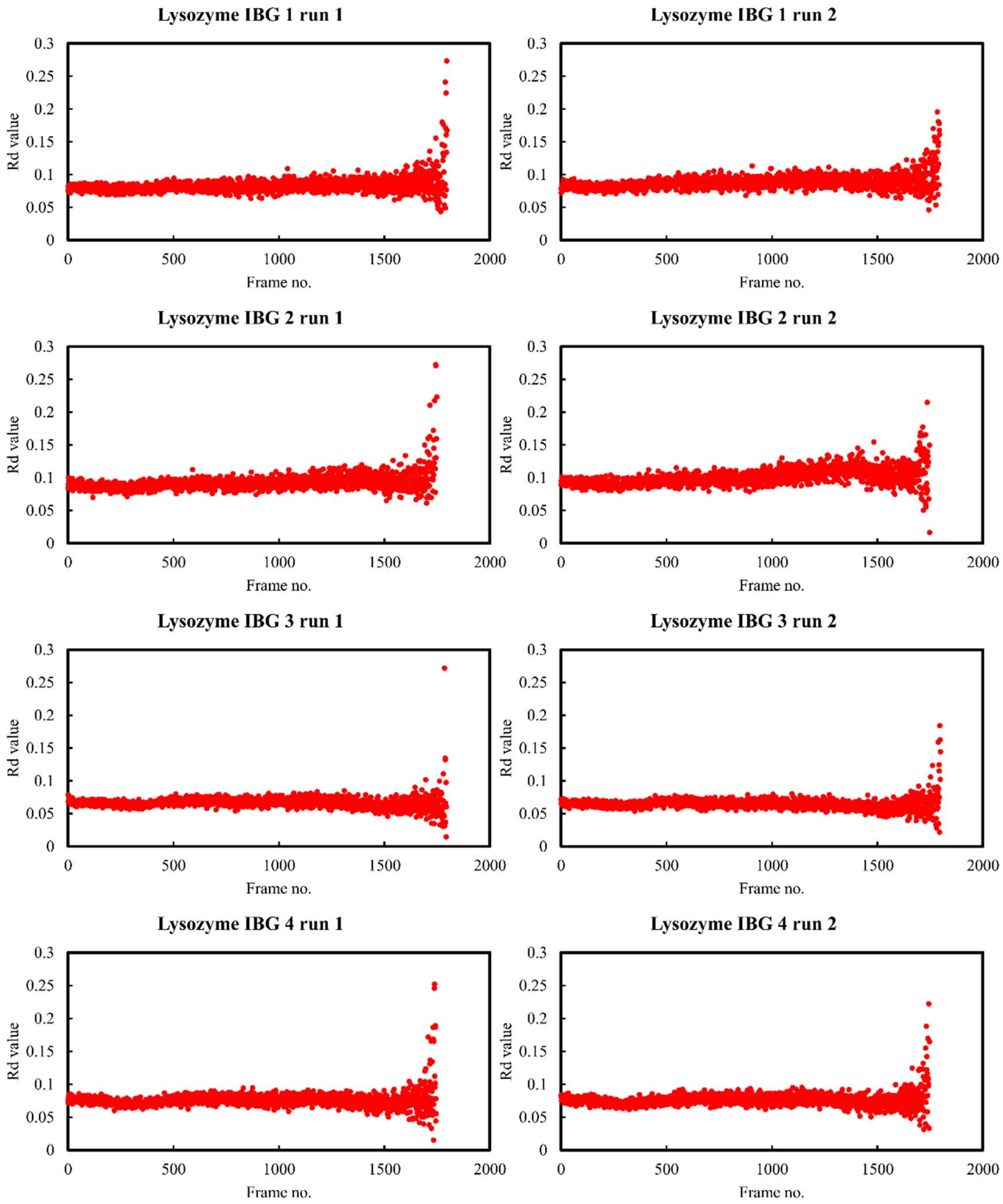


**Figure S2.** $R_D$ plots of the individual data sets of lysozyme IBG collection.

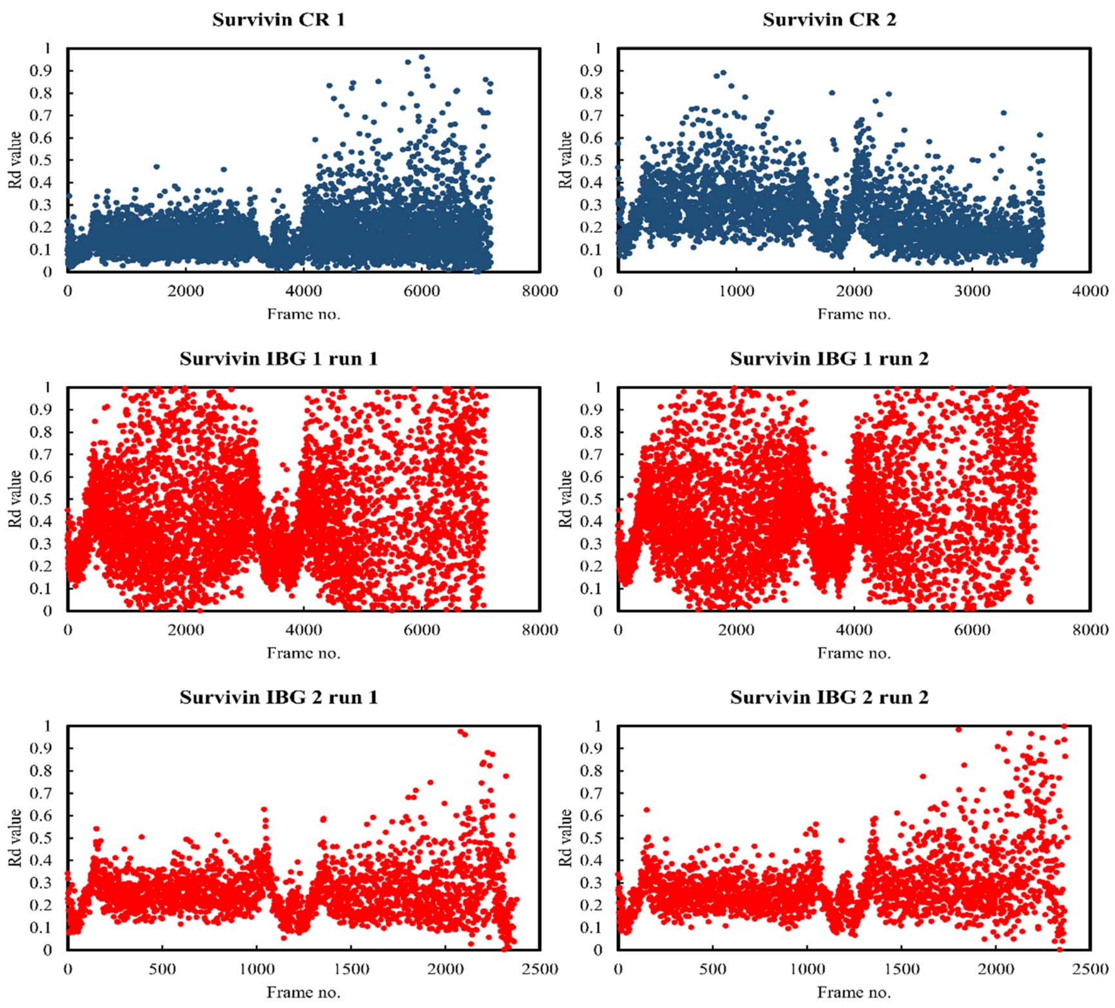


**Figure S3.** $R_D$ plots of the individual data sets of survivin CR (*blue*) and IBG (*red*) collection respectively.

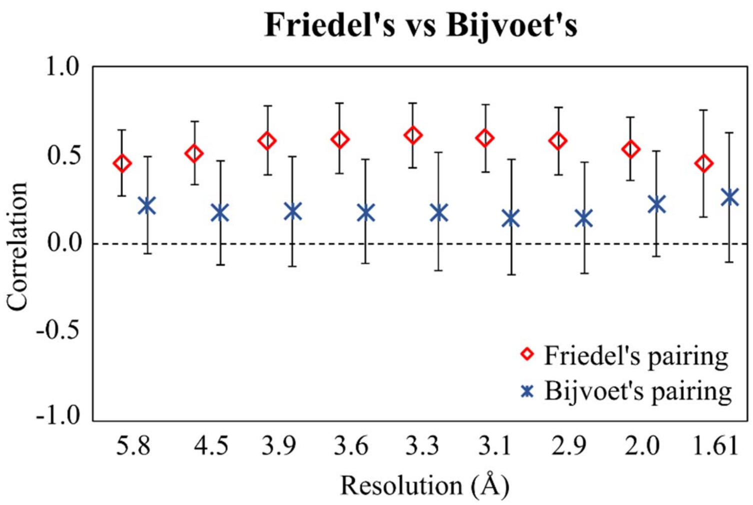


**Figure S4.** Pearson Coefficient correlation of lysozyme CR data set for Friedel pairing reflections (*red*) and the Bijvoet pairing reflections (*blue*) analyzed by multivariate Bayesian inference.

**Table S1.** Data collection parameters of the individual data sets in the order of collection time.

| Data set | Data collection starts | No images | Exposure time per frame (ms) | Oscillation range (º) | Starting X-ray flux | Detector distance (mm) | Estimated absorbed dose maximum (MGy) | Estimated average absorbed dose (MGy) |
|---|---|---|---|---|---|---|---|---|
| Survivin CR 1 | 14:21:49 | 7200 | 40 | 0.05 | $4.62 \times 10^{10}$ | 437 | 9.8 | 3.1 |
| Lysozyme CR 1 | 21:24:29 | 3600 | 40 | 0.10 | $2.37 \times 10^{10}$ | 155 | 4.0 | 0.44 |
| Lysozyme CR 2 | 21:35:06 | 3600 | 40 | 0.10 | $2.37 \times 10^{10}$ | 172 | 4.0 | 0.44 |
| Lysozyme CR 3 | 21:44:03 | 3600 | 40 | 0.10 | $2.38 \times 10^{10}$ | 191 | 4.0 | 0.45 |
| Lysozyme CR 4 | 21:49:48 | 3600 | 40 | 0.10 | $2.34 \times 10^{10}$ | 191 | 4.0 | 0.44 |
| Lysozyme CR 5 | 21:53:19 | 3600 | 40 | 0.10 | $2.32 \times 10^{10}$ | 191 | 3.9 | 0.44 |
| Lysozyme IBG 1 | 22:00:52 | 3600 | 40 | 0.10 | $2.27 \times 10^{10}$ | 191 | 3.8 | 0.43 |
| Lysozyme IBG 2 | 22:24:16 | 3600 | 40 | 0.10 | $2.28 \times 10^{10}$ | 191 | 3.9 | 0.43 |
| Lysozyme IBG 3 | 22:40:20 | 3600 | 40 | 0.10 | $2.28 \times 10^{10}$ | 191 | 3.9 | 0.43 |
| Lysozyme IBG 4 | 22:56:38 | 3600 | 40 | 0.10 | $2.31 \times 10^{10}$ | 191 | 3.9 | 0.44 |
| Survivin IBG 1 | 02:19:34 | 7200 | 40 | 0.05 | $3.88 \times 10^{9}$ | 469 | 0.82 | 0.26 |
| Survivin CR 2 | 03:04:10 | 3600 | 40 | 0.10 | $2.64 \times 10^{10}$ | 469 | 2.9 | 0.93 |
| Survivin IBG 1 | 03:16:26 | 4800 | 40 | 0.15 | $2.79 \times 10^{10}$ | 469 | 3.9 | 1.2 |

**Table S2.** Anomalous peak height comparison

| **Lysozyme** | | | | | **Survivin** | | | | |
|---|---|---|---|---|---|---|---|---|---|
| **Collection** | **CR** | | **IBG** | | **Collection** | **CR** | | **IBG** | |
| **Analysis** | **Univ.** | **Multiv.** | **Univ.** | **Multiv.** | **Analysis** | **Univ.** | **Multiv.** | **Univ.** | **Multiv.** |
| **Residue** | **Sigma** | | | | **Residue** | **Sigma** | | | |
| Cys24 | 15.1 | 15.4 | 15.6 | 15.5 | Zn 141 A | 24.0 | 20.4 | 12.2 | 13.7 |
| Met30 | 19.8 | 20.0 | 20.3 | 19.4 | Zn 141 B | 22.8 | 18.9 | 10.2 | 12.5 |
| Cys48 | 21.6 | 21.3 | 21.3 | 20.6 | | | | | |
| Cys82 | 23.9 | 22.9 | 22.0 | 21.4 | | | | | |
| Cys94 | 18.9 | 20.4 | 17.4 | 17.2 | | | | | |
| Cys98 | 22.0 | 22.5 | 22.2 | 21.6 | | | | | |
| Cys112 | 20.0 | 21.2 | 18.4 | 18.8 | | | | | |
| Met123 | 22.3 | 21.0 | 22.2 | 22.9 | | | | | |
| Cys133 | 20.4 | 20.0 | 19.5 | 18.9 | | | | | |
| Cys145 | 16.4 | 15.5 | 16.8 | 16.0 | | | | | |
| Cl154 | 11.5 | 11.1 | 11.8 | 11.5 | | | | | |
| Cl155 | 10.4 | 9.8 | 10.6 | 9.7 | | | | | |
| Cl156 | 7.2 | 6.5 | 6.7 | 7.4 | | | | | |
| Cl157 | 7.8 | 7.8 | 8.3 | 7.7 | | | | | |
| Cl158 | 15.7 | 15.6 | 14.9 | 14.5 | | | | | |
| Cl159 | 15.5 | 14.7 | 16.9 | 17.3 | | | | | |
| Cl160 | 23.0 | 22.1 | 23.2 | 22.8 | | | | | |
| Cl161 | 5.0 | 4.6 | 5.0 | 4.8 | | | | | |

**Table S3.** Phasing statistics of Bijvoet´s and Friedel´s pairing using the lysozyme CR data set treated by the multivariate Bayesian method.

| | **Lysozyme** | |
|---|---|---|
| Collection | **CR** | |
| Analysis | **Multiv. Bijvoet's pairs** | **Multiv. Friedel's pairs** |
| $R_{work}$ | 56.28 | 18.60 |
| $R_{free}$ | 58.70 | 20.14 |
| Nº frag. | 2 | 1 |
| Nº aa | 8 | 127 |
| Water | 15 | 138 |
| Sites | 7 | 18 |
| F. merit | 0.24 | 0.47 |
| PDB number | - | 6SIJ |
